# Comfort in palliative sedation (Compas): a transdisciplinary mixed method study protocol for linking objective assessments to subjective experiences

**DOI:** 10.1186/s12904-018-0316-2

**Published:** 2018-04-18

**Authors:** Stefaan Six, Steven Laureys, Jan Poelaert, Johan Bilsen, Peter Theuns, Reginald Deschepper

**Affiliations:** 1 Mental Health and Wellbeing research group, Vrije Universiteit Brussel, Laarbeeklaan 103, 1090 Jette, Belgium; 2 Coma Science group, Cyclotron Research Centre and Neurology Department, University and University Hospital of Liège, Avenue de l’hôpital 11, 4000 Liège, Belgium; 3 Department of Anesthesiology & Perioperative Medicine, Vrije Universiteit Brussel, Laarbeeklaan 103, 1090 Jette, Belgium; 4 Department of Experimental and Applied Psychology, Vrije Universiteit Brussel, Pleinlaan 2, 1000 Brussels, Belgium

## Abstract

**Background:**

In case of untreatable suffering at the end of life, palliative sedation may be chosen to assure comfort by reducing the patient’s level of consciousness. An important question here is whether such sedated patients are completely free of pain. Because these patients cannot communicate anymore, caregivers have to rely on observation to assess the patient’s comfort. Recently however, more sophisticated techniques from the neurosciences have shown that sometimes consciousness and pain are undetectable with these traditional behavioral methods. The aim of this study is to better understand how unconscious palliative sedated patients experience the last days of their life and to find out if they are really free of pain.

**Methods:**

In this study we will observe 40 patients starting with initiation of palliative sedation until death. Assessment of comfort based on behavioral observations will be related with the results from a NeuroSense monitor, an EEG-based monitor used for evaluation of the adequacy of anesthesia and sedation in the operating room and an ECG-based Analgesia Nociception Index (ANI) monitor, which informs about comfort or discomfort condition, based on the parasympathetic tone. An innovative and challenging aspect of this study is its qualitative approach; “objective” and “subjective” data will be linked to achieve a holistic understanding of the study topic. The following data will be collected: assessment of pain/comfort by the patients themselves (if possible) by scoring a Visual Analogue Scale (VAS); brain function monitoring; monitoring of parasympathetic tone; caregivers’ assessment (pain, awareness, communication); relatives’ perception of the quality of the dying process; assessment by 2 trained investigators using observational scales; video and audio registration.

**Discussion:**

Measuring pain and awareness in non-communicative dying patients is both technically and ethically challenging. ANI and EEG have shown to be promising technologies to detect pain that otherwise cannot be detected with the “traditional” methods. Although these technologies have the potential to provide objective quantifiable indicators for distress and awareness in non-communicative patients, strikingly they have not yet been used to check whether the current assessments for non-communicative patients are reliable.

**Trial registration:**

The study is registered on ClinicalTrials.gov (Identifier: NCT03273244; registration date: 7.9.2017).

## Background

Once death is imminent, a major concern of family members and caregivers is to assure maximal comfort for the patient during this terminal phase. This can often be achieved by ‘conventional’ pharmacological drugs such as opiates or other symptom-controlling drugs. However, when symptoms are refractory or very severe, e.g. suffocation, a more drastic option may be chosen, known as palliative sedation, terminal sedation or continuous deep sedation (CDS). In such cases comfort is sought by reducing the patient’s level of consciousness [1]. Notwithstanding palliative sedation is ethically controversial, this practice has substantially increased in hospitals, nursing homes and in the home setting. The overall reported incidences vary between 8% and 17% of all deaths [2–5]. Although CDS is more often used in palliative care settings, it is also applied in other populations such as coma patients and the demented elderly.

It is assumed that patients who are sedated according to current standards of care and the guidelines of CDS are unaware of their clinical situation and therefore do not experience symptoms of discomfort such as dyspnea, delirium, and other distressing conditions that are common during the terminal phase. However, a critical evaluation based on more recent evidence raises the question whether the current assessments of suffering and awareness are accurate. Three kinds of problems (assessment of (dis)comfort, the notion of (un)awareness, and titration of drugs) can be discerned.

### Problems with assessment of (dis)comfort in dying patients

The gold standard for detecting distress is patient self-reporting and several instruments, such as the Visual Analog Scale (VAS) or the Numeric Rating Scale (NRS) for pain, are based on this [6]. However, in the case of CDS, patients are unable to communicate whether or not they are still in distress or still (partially) aware of what is going on with them. Some scales have been developed for non-communicative patients as well, but several problems with those have been reported. One well-documented problem is that such scales seem unable to detect pain and awareness in all patients, e.g. because they depend on inferences made from patients’ motor responsiveness, which in case of CDS are nonexistent [7]. Another problem is that these scales are only partially, and in most cases not at all, validated for dying patients [8–10]. In the guidelines for palliative sedation it is acknowledged that the efficacy and safety of palliative sedation are not sufficiently understood and therefore it has been concluded that “there is no scale available to assess the patient’s comfort during sedation”. These findings cause an even bigger concern considering the evidence that family members of patients, compared with caregivers, often have different perceptions of a patient’s comfort and their quality of dying during CDS. While family members tend to overestimate pain, caregivers often seem to rather underestimate it [11]. Furthermore, interrater reliability among nurses and physicians often seems poor [12].

### Problems with (un)awareness

In recent years, doubts have risen on whether patients labeled ‘unconscious’ really are completely insensate and unaware. Studies in different types of patients and settings that critically reviewed awareness, consistently reported that persons were, in contrast to what was assumed by the caregivers, not always (completely) unaware. For example, several studies have shown that patients diagnosed with a vegetative state (currently also called ‘unresponsive wakefulness syndrome’) did show some (minimal) clinical signs of conscious awareness in about 40% of the cases [13]. In some cases, a purportedly unconscious patient could even reliably generate appropriate EEG responses to two distinct commands and occasionally was even able to establish basic communication with ‘yes’ or ‘no’ answers using functional Magnetic Resonance Imaging (fMRI) [14]. These findings demonstrate that at least a minority of clinically diagnosed unresponsive patients shows some residual cognitive function and conscious awareness and that even skilled caregivers were not able to recognize [13]. Also, patients with locked-in syndrome may be mistakenly considered unconscious as may some (rare) patients during general anesthesia [15]. In contrast to the setting where surgical or intensive care patients are managed, advanced monitoring equipment is usually lacking in palliative or home care settings. Terminal or palliative sedated patients ultimately die and therefore patient self-reporting is missing.

The above findings show that the ‘traditional’ clinical tools and procedures to assess comfort and awareness in dying non-communicative patients have important methodological limitations. It should be noted that the problems with assessments are not to be ascribed to a lack of competence in the caregivers, but are of a much more fundamental nature: the absence of reliable tools. The developers of guidelines are aware of these limitations. Although guidelines refer to some scales, the recent Belgian guideline rightly stresses that “their utility in palliative care is not proven” [16]. The current guidelines for CDS are limited to suggest “a daily visit of the physician” and to “continue attention for possible expressions of discomfort (e.g. facial expressions, movements…)” [3, 16].

### Problems with the titration of drugs

Since the aim of CDS is to give optimal comfort, but not to hasten death, the principle of ‘proportionality’ is a pivotal aspect of this treatment and hence the guidelines state that sedation should be ‘no deeper than necessary to avoid suffering’ [1, 16]. To meet this principle of proportionality, caregivers administer the doses of the drugs so that they are high enough to provide comfort but should not hasten death. Studies have shown that CDS does not usually affect survival time [17]. However, as palliative sedation is considered ‘slow euthanasia’ by some, physicians may be ‘extra careful’ with the use of high doses of sedative medication. Several studies have reported underuse of medicines due to a lack of knowledge, unwarranted beliefs, to avoid the perception of giving ‘excessive’ doses and even because of fear among caregivers of being accused of ‘killing’ the patient [18].

#### How to improve assessments of suffering?

Although measuring pain and awareness in non-communicative dying patients is challenging, it is possible. In recent years functional neuroimaging, like functional Magnetic Resonance Imaging (fMRI) and electroencephalography (EEG), have shown to be promising technologies to detect pain that otherwise cannot be observed or detected with the ‘traditional’ methods [14]. Although these technologies have the potential to provide objective quantifiable indicators for distress and awareness in non-communicative patients and to differentiate several types of discomfort (e.g., pain versus delirium), they have strikingly not yet been used to establish reliability of the current assessments for non-communicative patients. It is remarkable that, given the increasing incidence of CDS, there is so little concern about the possibility that patients may experience an uncomfortable dying phase while being unable to signal their suffering. An assessment tool that would allow clinicians to more accurately determine the appropriate doses of medications would encourage more vigorous symptom management in the dying.

Paradoxically, the inability to report distress might also be aggravated or become complete by the use of drugs that might abolish potential further communication and even facial expressions [19]. Hence, some patients might have subjective phenomenological awareness or suffering with very limited, fluctuating or absent behavioral motor signs of distress [20]. The fact that neuroimaging or electrophysiology recordings have not been used so far to validate the assessment tools for distress in non-communicative patients, even when doubts about these tools have arisen, may be related to the reluctance in palliative and end-of-life care to embarrass patients with high-tech equipments as in most cases, patients have already experienced a long treatment period.

In our view, we therefore urgently need a triangulation of methods by which existing scales, qualitative methods and neuroimaging are combined. Each method has its potential and limitations but combined they can be used to validate the commonly used observational scales. The COMPAS (COMfort in PAlliative Sedation) study protocol seeks to address this. As part of the grant application process by the Research Foundation - Flanders (FWO), the study design has been scientifically peer reviewed.

##### Aims of the study

- To better understand how unconscious palliative sedated patients experience the last days of their life.
- To find out if they are really free of pain.
- To evaluate to what degree assessments of comfort based on behavioral observations are in line with the results from a brain function monitor and a ANI-monitor (analgesia nociception index).
- Additionally we want to find out if changes in the measured depth of sedation can be experienced by the patient, caregivers and relatives, especially in the last moments of life when sometimes unexpected changes have been measured.

##### Research questions

1. Are sedated non-communicative dying patients free of pain and other kinds of discomfort?
2. Do the findings of the different assessment methods correspond to each other? In casu:
  - The commonly used assessments (observational scales)
  - The perceptions of family members
  - The neurophysiological measurements
3. Are there differences according to the setting? In casu:
  - Homes for the elderly
  - Hospitals (with Palliative Care Units (PCU) and/or Intensive Care Units (ICU))
4. What is the effect of contextual factors:
  - Medical and pharmacological factors (diseases, medical condition, feeding, medication, etc.).
  - Environmental factors (e.g. the presence of next of kin, noises etc.)

If one or more observational scales provide conclusive results that correspond to the results of the EEG and/or ANI measurement, this can be considered validation. In that case it would seem that observational scales can be reliably used in the palliative population. If not, then we may need to rethink our ways of measuring comfort in dying patients.

## Methods/design

Because of the complexity of the problem (discomfort is the result of many factors that, for ethical and practical reasons, researchers cannot control) and the explorative nature of this study, it would be conceived as a multi-case study in which the setting and participants are deliberately chosen. The aim is not to achieve representativity, but rather to describe some typical cases which can provide maximum insight.

### Participants

In modal hospitals and modal homes for the elderly, forty patients will be selected. In each setting 20 participants will be included consecutively, which should result in 40 detailed case studies. Patients will be deliberately selected to gain maximum insight and reflect variability regarding medical conditions.

#### Inclusion criteria

Patients may be included if they are considered by their treating physician as:

1. in their last week of life
2. in conditions that might, when not treated, cause high levels of distress
3. palliative sedation will be started

The treating physicians (specialists at the PCU/ICU or general practitioners at homes for the elderly) will be asked to determine whether or not the patients meet these conditions by means of a checklist on these 4 criteria. To optimally reflect daily practice, no further specific instructions will be given on how to evaluate these criteria and treatment will be according to the physician’s judgment and the best practice.

### Procedures

For this prospective study methods from social sciences (qualitative data collection based on interviews, observation), quantitative assessments (scales to assess awareness and discomfort) and neurophysiological data will be combined. Hence, a combination of methods that can be considered fairly objective (e.g. EEG and physiological parameters) and rather subjective methods (e.g. observations by family members) will be used to assess discomfort and awareness. Therefore, the following data will be collected:

*1. Assessment by caregivers* (physicians and nurses) in the routine manner. As explained above, this can be based either on their ‘experience’ or on tools they routinely use in their practice. Every day all treating physicians and nurses will be asked to fill in a brief questionnaire (3 VAS scales) on the patient’s level of awareness (no awareness- completely aware), comfort (no pain – very severe pain) and ability to communicate (no communication possible - full communication possible). After the patient has deceased, all caregivers will be interviewed and asked to comment on their assessments.

*2. Family members’ perception* of the quality of the dying process, their opinion about the patient’s comfort and awareness and their impression of whether they had any kind of (non-verbal) contact with the patient. Parallel with the above described procedure, family members will also be asked to fill in the same 3 VAS scales. Semi-structured interviews will be conducted before and after the patient has deceased.

*3. Assessment by 2 trained investigators* using 1 scale that is mentioned in the Flemish palliative sedation guideline and 3 other scales that have been proposed in the literature [16]:

3.1 CCPOT (Critical Care Pain Observational Tool), a tool specifically developed for use in patients with limited consciousness [8].
3.2 RASS (Richmond Agitation-Sedation Scale) [21].
3.3 M-ESAS (Modified Edmonton Symptom Assessment Scale, validated for a Flemish Palliative Care Population) [22].
3.4 BPS-NI (Behavioral Pain Scale Non-Intubated) [23].

*4. Neurophysiological assessments* of distress and level of awareness using fMRI has been successfully used to detect awareness and distress that could not be detected by thorough behavioral assessment [10, 13, 14, 24, 25]. Issues of availability and extra stress incurred by patients when transferred are making the use of fMRI for practical and ethical reasons not feasible.

EEG has recently been shown to provide a feasible and methodologically sound alternative for bedside detection of awareness and pain [26–28]. Although results from these methods need to be interpreted with caution, they can provide important indicators that are especially valuable for assessments in non-communicative patients. Data will be collected with a Neurosense monitor that can easily be used and set up. It displays two frontal EEG signals, and calculates a number of parameters including the bilateral WAVcns index (Wavelet Anesthetic Value for Central Nervous System), ranging from 100 (awake) to 0 (flat EEG). The lower the index, the lower the likelihood of consciousness. The recommended WAVcns for general anesthesia with low probability of consciousness is between 60 and 40. For technical details on the respective measurements of awareness and distress we refer to Schulz E et al. and Cruse D et al. [24, 29].

Additionally, heart rate variability (HRV) will be measured using an ANI monitor. HRV is the variation in time intervals between heartbeats and reflects the effect of the vagus nerve on the heart, which is inhibited during pain. It has already been widely studied in relationship to pain, and seems to be a promising biomarker [30]. However, studies with palliative sedated patients show contradicting results thus far [31, 32]. A promising non-invasive technique is the continuous monitoring of HRV transformed into an analgesia nociception index (ANI, 0–100), which assesses parasympathetic activity as a possible measure of nociception [33]. The ANI provides greater stability than raw indices of HRV. A recent study showed that ANI is effective in detecting pain in deeply sedated critically ill patients [34]. The analgesia nociception index is a non-invasive tool based on the analysis of the respiratory fluctuations of heart rate that mainly reflect the variability in the parasympathetic tone. The NeuroSense and ANI monitor record the EEG and ECG signal continuously, enabling a quantitative assessment of these parameters. The data can be used to assess the level of sedation but also to register negative and positive emotion-related activities such as the voice of loved ones and distress.

*5. Observation* of the patient to detect e.g. restlessness, movements etc. To assure a more precise and reliable observation, video and audio registrations will be made of the patient.

*6. Background information* based on the patient’s medical file, observation and interviews.

6.1 Characteristics of the patient (age, gender, profession…).
6.2 Medical information: history, medical condition, feeding, medication, etc.).
6.3 Pharmacological information (especially medication aimed to improve comfort e.g. sedatives, painkillers etc.)
6.4 Environmental information (e.g. the presence of next of kin, noises etc.)

Data will be collected every day, starting from the day when palliative sedation is initiated until the patient dies. Neurophysiological data will be collected continuously. Video and audio registrations will preferably take place non-stop (in consultation with the family) but may be interrupted during family visits to respect privacy.

### Sample size

A previous study learns that a correlation of 0.45 was found between Bispectral Index (BIS) monitoring and the Ramsay scale [35]. A similar correlation can be expected between the WAVcns and the VAS in the present study. However, in order to become a successful measure of sedation, we require the correlation between both measures to be significantly higher than 0.30. Although EEG monitoring would allow continuous data collection, the VAS will be completed only once or twice a day by relatives (more often by trained investigators) to keep burden as low as possible. As we expect patients can be monitored during 2–3 days on average, some 5 VAS scores (from relatives, more from staff) will be available per patient. This results in a close to 80% probability for our study to find a correlation higher than 0.30 if the actual correlation is 0.45.

### Data-analysis

Analysis of qualitative data (interviews and video) will be done in accordance with the interpretative paradigm, aiming to understand meanings and actions and how people construct them, and follow the principles of Grounded Theory [36]. Coding of the neurological data will be performed by an experienced neurologist.

Video registrations will be coded in events and analysed using nVivo, a tool for qualitative analysis that enables researchers to code and analyze events and also allows importing and correlating video and neurophysiological data. A coding scheme will be developed to code behavioral signs of the patient, treatments (painful acts, medical acts, etc.) and environmental circumstances and changes (visits, noises…).

The involved researchers will intensively cooperate in the interpretation of the data. All data will be used for an overall assessment (using the transdisciplinary mixed methods case study approach) [37]. The analysis will focus on:

- comparison of the results based on the different methods
- detection of changes in level of comfort and awareness

The findings and interpretations will then be discussed in a multidisciplinary team consisting of members of the Mental Health and Wellbeing Research Group, the department of Anesthesiology and Perioperative Medicine, the department of Experimental Psychology (all from the Vrije Universiteit Brussel) and the Coma Science Group (University of Liège).

### Ethical aspects

Video/audio registration will only be made after explicit consent of the patient (if possible) and his next-of-kin.

The study protocol is approved by the biomedical ethics committee of the VUB/UZ Brussel (BUN 14320136504) and additional approval will be asked from participating hospitals. Written informed consent will be asked from the patient or his/her substitute decision maker.

We are aware that the data collection is challenging and delicate, especially because we will deal with dying patients and their family. Therefore, special attention will be given to clear communication about the aims of our study. We will explain participants that the study is not invasive and will not hinder the (sedated) patient and that the aim is to minimize the risk for suffering.

## Discussion

The objectives of the COMPAS study are to determine if more objective measures based on EEG and HRV are potentially useful in the assessment of comfort and pain during palliative sedation. To achieve these goals a transdisciplinary mixed methods multiple case study design is adopted, where both quantitative and qualitative data are being linked to neurophysiological data collected by monitoring devices. The methodological background of this particular type of mixed methods research has been described by Deschepper et al. in the paper *Linking numbers to perceptions and experiences: why we need transdisciplinary mixed-methods combining neurophysiological and qualitative data* [37]. Next to the already mentioned idiosyncratic aims the present study also aspires to be the first worked example of this methodological approach.

### Strengths

To our knowledge this is one of the very few studies which seek to take an in-depth view on the assessment of pain and discomfort in non-communicative palliative sedated patients. To this effect multiple ways of data collection are used, in particular both objective and subjective assessments, with the intent to develop as complete a picture as possible of the subject at hand. By using a transdisciplinary approach throughout the entirety of the study, a high degree of integration is achieved; researchers from different disciplines were involved in all aspects of the study, as were members of the palliative community, patients and family members to decide what is ethically and practically acceptable.

### Limitations

There are a number of limitations to this study, partly because doing research at the end of life is delicate, and partly because of the methodological challenges in dealing with non-communicative unconscious patients. It is likely that more extensive measures of brain activity such as fMRI would yield more sophisticated data, but, more extensive neurophysiological monitoring would be more invasive as compared to a NeuroSense monitor and this would likely be more disturbing for family members.

From a methodological point of view, the greatest challenge will be how to combine the different types of data (neurophysiological, quantitative and qualitative) to assess a phenomenon which is considered to be best assessed by self-reporting. Since the latter is not an option, the best possible way to approximate objectivity is by triangulation, combining different measures that are collected at the same moment and analyzed transversally. However gathering different types of data (e.g. assessments of family members) may prove difficult in such a sensitive setting.

### Opportunities

Completion of this project will result in a deeper understanding of how the assessment of (dis)comfort and pain during palliative sedation may be potentially improved by including more objective neurophysiological parameters. Additionally, our findings will clarify if the present practices associated with assessing patient comfort during palliative sedation (based on observational scales) need to be reconsidered. The transdisciplinary approach of the researchers involved in the COMPAS study further ensures a maximum level of ecological validity when studying such complex phenomenon.

### Threats

The threats to the successful conduct of this study relate mainly to the reluctance of the patient or his substitute decision maker to be bothered with participating in a study at such a sensitive and difficult moment. After all, one of the ideas behind the palliative care concept is to give comfort to the patient and that includes an environment that feels more natural and home-like, so the concept of using monitoring devices in such a setting seems contra-indicated. This concern has been voiced by palliative caregivers. However, we feel confident that with proper and sensible explanation the importance of this study can be made understood. Furthermore every aspect of the study has been designed to be as minimally burdensome as possible. Already a pilot case has demonstrated the feasibility of this design for all actors involved, the protocol for this observational study has been registered retrospectively at ClinicalTrials.gov (ID NCT03273244) [38].

## Abbreviations

ANI: analgesia nociception index
CDS: continuous deep sedation
COMPAS: COMfort in PAlliative Sedation
EEG: electroencephalography
fMRI: functional Magnetic Resonance Imaging
HRV: heart rate variability
ICU: intensive care unit
NRS: Numeric Rating Scale
PCU: palliative care unit
VAS: Visual Analog Scale
WAVcns: Wavelet Anesthetic Value for Central Nervous System
